# Protease associated domain of RNF43 is not necessary for the suppression of Wnt/β-catenin signaling in human cells

**DOI:** 10.1186/s12964-020-00559-0

**Published:** 2020-06-11

**Authors:** Tomasz Radaszkiewicz, Vítězslav Bryja

**Affiliations:** 1 Department of Experimental Biology, Faculty of Science, Masaryk University, Brno, Czech Republic; 2 Department of Cytokinetics, Institute of Biophysics, Academy of Sciences of the Czech Republic, Brno, Czech Republic

**Keywords:** RNF43, Protease associated domain, Wnt signaling, LRP6, RSPO1, Dishevelled

## Abstract

**Background:**

RNF43 and its homolog ZNRF3 are transmembrane E3 ubiquitin ligases frequently mutated in many human cancer types. Their main role relays on the inhibition of canonical Wnt signaling by the negative regulation of frizzled receptors and LRP5/6 co-receptors levels at the plasma membrane. Intracellular RING domains of RNF43/ZNRF3 mediate the key enzymatic activity of these proteins, but the function of the extracellular Protease Associated (PA) fold in the inhibition of Wnt/β-catenin pathway is controversial up-to date, apart from the interaction with secreted antagonists R-spondin family proteins shown by the crystallographic studies.

**Methods:**

In our research we utilised cell-based approaches to study the role of RNF43 lacking PA domain in the canonical Wnt signalling pathway transduction. We developed controlled overexpression (TetON) and CRISPR/Cas9 mediated knock-out models in human cells.

**Results:**

RNF43ΔPA mutant activity impedes canonical Wnt pathway, as manifested by the reduced phosphorylation of LRP6, DVL2 and DVL3 and by the decreased β-catenin-dependent gene expression. Finally, rescue experiments in the CRISPR/Cas9 derived *RNF43*/*ZNRF3* double knock-out cell lines showed that RNFΔPA overexpression is enough to inhibit activation of LRP6 and β-catenin activity as shown by the Western blot and Top flash dual luciferase assays. Moreover, RNF43 variant without PA domain was not sensitive to the R-spondin1 treatment.

**Conclusion:**

Taken together, our results help to understand better the mode of RNF43 tumor suppressor action and solve some discrepancies present in the field.

Video Abstract

**Graphical Abstract:**

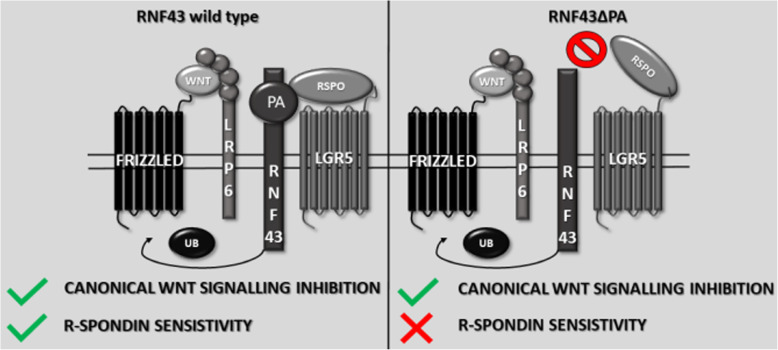

## Background

RING finger protein 43 (RNF43) and its homolog Zinc/Ring finger protein 3 (ZNRF3) are E3 ubiquitin ligases belonging to the PA-TM-RING family [1]. These enzymes bear a resemblance to the typical type I transmembrane receptors. Their extracellular N-terminal part contains signal peptide followed by the protease-associated (PA) domain, while catalytic Really Interesting New Gene (RING) zinc finger domain is found in the intracellular C-terminal fragment [2].

RNF43/ZNRF3 enzymes are recently being extensively studied because of their involvement in the various aspects of cellular biology and carcinogenesis. RNF43 and ZNRF3 are negative regulators of the Wnt signaling pathway, which is a crucial signaling axis controlling various aspects of the development and disease (for more details reader is encouraged to check available reviews, i.e. [3–5]). RNF43 and ZNRF3 act as tumor suppressors and numerous mutations in the *RNF43* gene were identified in cancers of various tissues, like endometrium, stomach, ovary, pancreases or colon [6–9]. The main molecular function of RNF43/ZNRF3 in the Wnt pathway is the negative regulation of the surface level of Wnt receptors Frizzled (FZD) and co-receptor low-density lipoprotein receptor-related protein 6 (LRP6) by their ubiquitination and subsequent degradation [10, 11]. Additionally, it was proposed that RNF43 can act downstream from the plasma membrane receptor complexes by tethering the T-cell factor 4 (TCF4) to the nuclear membrane, preventing gene transcription [12].

The importance of enzymatic activity of the RING domain for RNF43/ZNRF3 ability to inhibit Wnt pathway is undoubtful. It was proven that point mutations disrupting this catalytic domain had dominant negative effect [10]. Region adjacent to the RING domain was also shown to interact with dishevelled (DVL) protein, mediating RNF43/ZNRF3 dependent frizzled degradation [13]. These observations underline the crucial role of the intracellular RNF43/ZNRF3 proteins regions in the facilitating signaling events. On the other side, the function of RNF43/ZNRF3 ectodomain, in particular PA domain, remains under the debate. It seems to be clear that PA domain mediates the interaction with R-spondins (RSPO)- endogenous negative regulators of RNF43 and ZNRF3. RSPO 1–4 are secreted proteins, which reduce plasma membrane level of RNF43 and ZNRF3 in a leucine-rich repeat-containing G-protein coupled receptors (LGR) 4/5/6 dependent (RSPO1 and 4) or independent (RSPO2 and 3) way [14]. Crystal structures revealed that R-spondins bind PA domain of RNF43 and ZNRF3 and form the ternary complex with LGR4/5/6 [15–20].

It is currently unclear whether PA domain is required for binding to FZD and RNF43/ZNRF3-mediated inhibition of Wnt/β-catenin pathway. Experiments in *Caenorhabditis elegans* showed that PLR-1ΔPA mutant, which is *C. elegans* homolog of RNF43 and ZNRF3*,* was not able to reduce surface level of MIG-1/FZD and block Wnt signaling [21]. Also, MIG-1/FZD deprived of cysteine-rich domain (CRD) was unaffected upon PLR-1 overexpression. Next, experiments established in the mammalian cells showed that deletion of the whole extracellular part of the RNF43 prevented RNF43-mediated FZD5 internalization [10]. Moreover, another group described that precise deletion of the PA domain of RNF43 blocked its inhibitory function on the β-catenin dependent transcription and only part of *Xenopus* embryos injected with *RNF43ΔPA* mRNA showed phenotype similar to the observed for the wild type *RNF43* [22]. The same study demonstrated interaction between PA domain of RNF43 and CRD of FZD5 in the co-immunoprecipitation assay after overexpression [22]. However, other researchers did not succeed to positively verify the existence of this interaction [13]. Similarly, binding of ZNRF3 to the CRD domain of FZD8 was not detected in a surface plasmon resonance based binding assay [18].

The existence of the above described discrepancies encouraged us to look at the role of RNF43 PA domain in the negative regulation of canonical Wnt signaling in detail. In order to shed light on the mechanism of RNF43/ZNRF3 function and regulation, we generated several novel mammalian models to study consequences of PA deletion in the cell-based assays. Our data collectively suggest that PA domain is dispensable for RNF43 capacity to block Wnt signaling pathway.

## Methods

### Cell lines and treatments

T-REx 293 (R71007, Thermo Fisher Scientific) cells and all their derivates were cultured at 37 °C and under controlled 5% (vol/vol) CO_2_ atmosphere in the Dulbecco’s modified Eagle’s medium (DMEM, 41966–029, Gibco, Life Technologies) supplemented with 10% fetal bovine serum (FBS, 10270–106, Gibco, Life Technologies), 2 mM L-glutamine (25,030,024, Life Technologies) and 1% penicillin-streptomycin (XC-A4122/100, Biosera).

Endogenous Wnt ligands secretion was blocked by the use of 0.5 μM LGK-974 Porcupine-specific inhibitor (1,241,454, PeproTech). For the purpose of canonical Wnt pathway stimulation, cells were treated with the recombinant human WNT3A (rWNT3A) (CF 5036-WN-CF, RnD Systems) for 3 h or overnight for the Top flash dual luciferase assay in the indicated concentrations (40–100 ng/ml). Recombinant RSPO1 (rRSPO1) (120–38, PeproTech) in the final concentration of 25, 50 or 100 ng/ml was employed as co-treatment with rWNT3A for the canonical Wnt pathway activation enhancement. Inducible protein expression was activated by the overnight, if not specified otherwise, tetracycline treatment in the dose of 1 μg/ml (60–54-8, Santa Cruz Biotechnology).

### Plasmids, cloning and mutagenesis

For the purpose of RNF43 and RNF43ΔPA T-REx 293 inducible cell lines generation, cDNA encoding human *RNF43* fused with BirA* and HA tags was amplified by the Phusion High Fidelity DNA polymerase (F530S, Thermo Fisher Scientific) based PCR reaction and cloned into the HindIII (ER0501, Thermo Fisher Scientific) and XbaI (ER0681, Thermo Fisher Scientific) linearized pcDNA4/TO backbone (Thermo Fisher Scientific) by the In-Fusion cloning method (Takara Bio). As a result, pcDNA4-TO-RNF43-BirA*-HA plasmid was obtained. Next, the same vector was used as a template for generation of the RNF43 mutant lacking PA domain - pcDNA4-TO-RNF43-ΔPA-BirA*-HA. In-frame deletion of amino acids at positions no. 87–186 was performed using QuikChange II XL site-directed mutagenesis kit (Agilent) according to the manufacturer’s instructions. All produced plasmids were validated by the Sanger sequencing method.

For targeting RNF43 and ZNRF3 in the T-REx 293, gRNAs coding sequences: TGAGTTCCATCGTAACTGTG*TGG* (*PAM*) and AGACCCGCTCAAGAGGCCGG*TGG* were cloned into the gRNA-GFP-T1 backbone using previously described protocol [23].

Other plasmids used in this study are: pCAG-mGFP (Addgene #14757 [24]), pRLtkLuc (Promega), Super8X TOPFLASH (gift from Randall Moon), hCas9 (Addgene #41815 [25]), gRNA_GFP-T1 (Addgene #41819 [25]), pcDNA4-TO-RNF43Mut1-2xHA-2xFLAG [10], PiggyBack-Hygro and Transposase encoding plasmids (kindly gifted by Bon-Kyoung Koo). Primers sequences are shown in the Table 1.

**Table 1:** Primers

Cloning and mutagenesis primers			
Primer	Sequence		Purpose
RNF43 InFusion F	GTTTAAACTTAAGCTTATGAGTGGTGGCCACCAG		RNF43-BirA*-HA into pcDNA4
RNF43 InFusion R	AAACGGGCCCTCTAGACTATGCGTAATCCGGTACA		
del87–186	ATTAATGCAGTCCCACGAGCTGAAGGAGCCCC		Site-directed mutagenesis, deletion of the PA domain
del87–186-antisense	GGGGCTCCTTCAGCTCGTGGGACTGCATTAAT		
**Quantitative Polymerase Chain Reaction**			
GENE	Forward primer seq.	Reverse primer seq.	Product size
*B2M*	CACCCCCACTGAAAAAGATG	ATATTAAAAAGCAAGCAAGCAGAA	167
*GAPDH*	GACAGTCAGCCGCATCTTCT	TTAAAAGCAGCCCTGGTGAC	127
*RNF43*	TTTCCTGCCTCCATGAGTTC	CAGGGACTGGGAAAATGAATC	116
*ZNRF3*	GCTTTCTTCGTCGTGGTCTC	GCCTGTTCATGGAATTCTGAC	91
*CTNNB1*	AAAGCGGCTGTTAGTCACTGG	CGAGTCATTGCATACTGTCCAT	215
*AXIN2*	TACACTCCTTATTGGGCGATCA	TTGGCTACTCGTAAAGTTTTGGT	151
**CRISPR/Cas9 Genotyping**			
Primer	Sequence		Purpose
*RNF43* F	AGGTCTTTTACAAGTAGATAATAGCAAGG		CRISRP/Cas9 *RNF43* RFLP analysis
*RNF43* R	GCTGCAACCCTTTCCCA		
*ZNRF3* F	TTCTCAGCCTTGCTCTTCTG		CRISRP/Cas9 *ZNRF3* RFLP analysis
*ZNRF3* R	TCACCCTGGCCTTGAGTC		

### Transfections and stable cell lines generation

T-REx 293 cells were transected using 1 μg/ml, pH 7.4 polyethylenimine (PEI) and plasmid DNA in 4:1 ratio, applying already described method [26]. For the Top flash dual luciferase assay and immunofluorescence imaging, cells growing on 24 well plate were transfected by 0.4 μg of total plasmid DNA per one well. For stable cell lines preparation, 6 μg of pDNA was used for plasmids introduction into the cells growing on 10 cm plates. 24 h post transfection, cells were further processed and analyzed, or culture medium was changed for the fresh one, containing proper antibiotics for selection of positively transfected clones. pcDNA4/TO backbone encodes blasticidin S resistance (used 5 μg/ml, 3513-03-9, Santa Cruz Biotechnology) and PiggyBack-Hygro – hygromycin B (200 μg/ml, 31,282–04-9, Santa Cruz Biotechnology). Empty backbone pcDNA3.1 (Invitrogen) was used to equalize total plasmid DNA amount.

### Top flash dual luciferase assay

Canonical Wnt signaling activation leads to the β-catenin stabilization, allowing its translocation and changes in the gene expression. To measure the transcriptional activity of the β-catenin, cells were transfected with 0.1 μg of the pRLtkLuc plasmid and 0.1 μg of the Super8X TopFlash and subsequently treated overnight with rWNT3A, rRSPO1 in the presence of 0.5 μM LGK-974 and tetracycline, as marked in the figures. Approximately 24 h post-transfection, cells were washed with PBS and freezed. Subsequently, assay was performed according to the manufacturer protocol (E1960; Promega) and luminescence was detected by the use of Hidex Bioscan Plate Chameleon Luminometer. Results are presented as ratios of firefly (containing Wnt-specific TCF/LEF binding sites in the promoter) to renilla (constitutively active promoter) luciferases measurements, RLU - relative luciferase units.

### CRISPR/Cas9 mediated gene editing

For targeting *RNF43* and *ZNRF3* genes in the T-REx 293 cells, gRNAs: TGAGTTCCATCGTAACTGTG*TGG* (*PAM*) and AGACCCGCTCAAGAGGCCGG*TGG* were cloned into the gRNA_GFP-T1 backbone and co-transfected with PiggyBack-Hygro and Transposase coding plasmids using PEI. Then, cells were hygromycin B selected and plated as single cells on 96-well plates. For identification of clones carrying CRISPR/Cas9-mediated modifications in the sequences of both alleles, RFLP analysis using restriction enzymes was performed. Genomic DNA isolation from grown clonal cell lines was performed using DirectPCR Lysis Reagent (Cell) (Viagen Biotech), supplemented with 0.5 mg/ml Proteinase K (EO0491, Thermo Fisher Scientific). DreamTaq DNA Polymerase (EP0701, Thermo Fisher Scientific) based PCR was done according to the manufacturer instruction for amplification of gDNA fragment containing targeted sequence. PCR products were analyzed by restriction digestion, using Taal (ER1361, Thermo Fisher Scientific) for *RNF43* and HpaII (ER0511, Thermo Fisher Scientific) for *ZNRF3,* for detection of Cas9 mediated disruption of the recognition sites nearly the PAM sequences. Finally, all clones were sequenced using the Illumina platform and compared with the reference sequence [27]. Predictions of amino acid sequence changes due to the frameshift type mutations and occurrence of premature STOP codons were done. Primers sequences are shown in the Table 1.

### Western blotting and antibodies

Western blot method was done accordingly to the previously published protocol [28]. In brief, cells were washed with PBS and subsequently lysed in the sample buffer (2% SDS, 10% glycerol, 5% β-mercaptoethanol, 0.002% bromphenol blue and 0.06 M Tris HCl, pH 6.8), sonicated and thermally denatured. Electrophoretically separated proteins, in the SDS-PAGE gels, were transferred onto Immobilon-P PVDF Membrane (IPVH00010, Millipore) and detected by primary and corresponding HRP-conjugated secondary antibodies on Fusion SL imaging system (Vibler), using Immobilon Western Chemiluminescent HRP Substrate (Merck, WBKLS0500). Western blot signals were quantified using ImageJ software**.** Antibodies are listed in the Table 2. Western blot membranes are presented together with molecular mass markers in the Additional file 1.

**Table 2:** Antibodies

Antibody	Manufacturer	Dilution and application	Reference
LRP6 (C47E12)	cs-3395, Cell Signaling Technology	1:1000, WB	[28]
LRP6 (Phospho-S1490)	cs-2568, Cell Signaling Technology	1:1000, WB	[28]
β-actin	CS-4970, Cell Signaling Technology	1:3000, WB	[26]
DVL-2	CS-3216, Cell Signaling Technology	1:1000, WB	[26]
DVL-3	CS-3218, Cell Signaling Technology	1:1000, WB	[26]
HA-11	MMS-101R, Covance	1:2000 WB; 1:500, IF	[28]
a-mouse IgG HRP	A4416	1:4000 WB	–
a-rabbit IgG HRP	A0545	1:4000 WB	–
a-mouse Alexa Fluor™568	A10037, Thermo Fisher Scientific	1:600 IF	–

### Immunofluorescence and confocal microscopy

For imaging of the plasma membrane localization of ectopically expressed RNF43 and its PA domain lacking mutant, T-REx 293-RNF43-TetON and T-REx 293-RNF43ΔPA-TetON cells were grown on coverslips coated with 0.1% gelatin in PBS (Sigma) and 1 μg/ml human fibronectin (1918-FN-02, RnD Systems). At approximately 70% of confluence, they were transfected with plasmid pCAG-mGFP encoding membrane-bound form of GFP fused with a palmitoylation sequence of GAP43 at its N-terminus. Approximately 8 h after transfection, cells were stimulated with tetracycline for transgenes expression induction. Next day, cells were washed by PBS, fixed in 4% paraformaldehyde for 20 min, permeabilized by 0.1% Triton X-100 in PBS and blocked in the 2% solution of bovine serum albumin in PBS. Then, samples were incubated overnight at 4 °C with primary anti HA tag antibodies diluted in 2% BSA in PBS and washed. Corresponding Alexa Fluor secondary antibody (Invitrogen) was incubated with samples for 1 h at the room temperature, along with 0.5 μM of TO-PRO-3 Iodide (642/661) (T3605, Thermo Fisher Scientific) for nuclei counterstaining. After PBS washes, coverslips were mounted in the DAKO mounting medium (S3023, DAKO). Images were taken on the confocal laser scanning microscopy platform Leica TCS SP8 (Leica). Histograms of signals in each channel were prepared in the LAS X Life Science (Leica) software and plotted in the GraphPad Prism 8 for the co-localization analysis. Antibodies used are listed in the Table 2.

### Quantitative real-time polymerase chain reaction (qPCR)

To assess the expression level of *RNF43*, *ZNRF3*, *CTNNB1* and *AXIN2* (canonical Wnt signaling target gene [29]) in the response to the various treatments (rWNT3A 100 ng/ml and rRSPO1 50 ng/ml), qPCR reaction was applied. mRNA was extracted from cells using RNeasy Mini Kit (74,106; Qiagen) according to the manufacturer’s instructions. One microgram of mRNA was transcribed to the cDNA by RevertAid Reverse Transcriptase (EP0442, Thermo Fisher Scientific) and analyzed by use of a LightCycler® 480 SYBR Green I Master (04887352001, Roche) and Light Cycler LC480 (Roche). Results are presented as 2^−ΔΔ*CT*^ and compared by unpaired Student’s *t* test. Mean expression of *B2M* and *GAPDH* was used as reference. Primers sequences are shown in the Table 1.

### Statistics

Statistical significance was confirmed by paired or unpaired Student’s *t* test. *P* value < 0.05 was used to determine statistically significant difference. Statistical analysis and data visualization were performed in GraphPad Prism 8.0 software. All graphs are presented as mean ± SD.

## Results

### Inducible RNF43 and RNF43ΔPA expression in T-REx 293 cell line

**Fig. 1:**
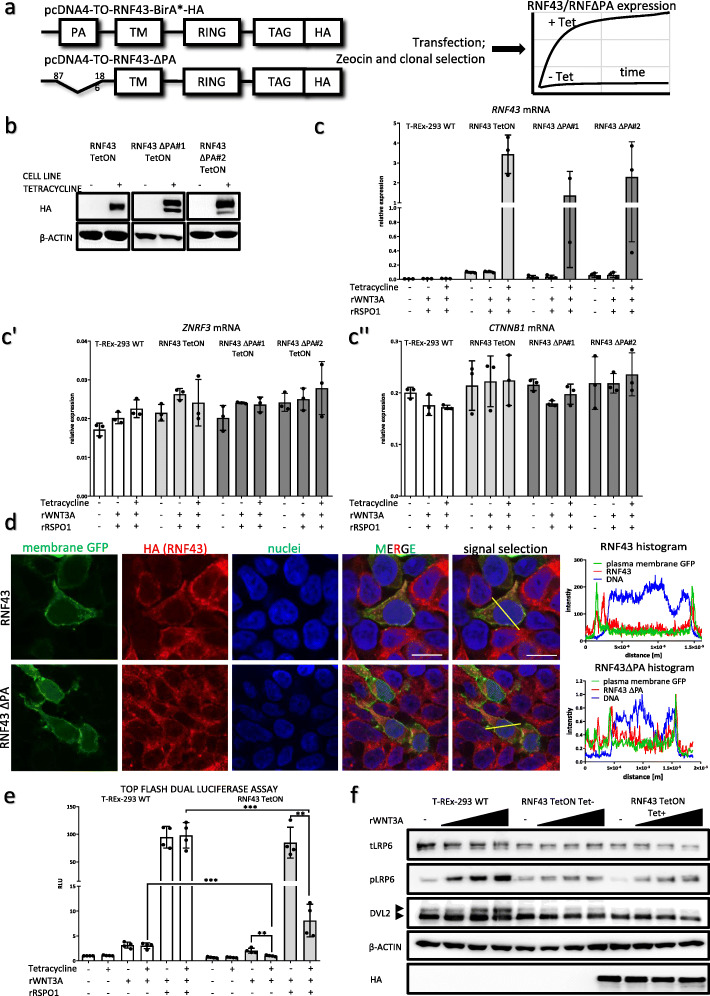
Inducible RNF43 and RNF43ΔPA expression in T-REx 293 cell line. **a.** Schematic representation of the used experimental models. T-REx 293 cell lines inducibly expressing tagged wild type RNF43 or its variant lacking PA (aa 87–186) domain. Supplementation of culture medium with Tet drives transgene expression. **b.** Western blot showing Tet-induced expression of HA-tagged RNF43 constructs in stable sell lines. β-actin served as loading control. **c.** Expression of *RNF43* (c)*, ZNRF3* (c’) and *CTNNB1* (c”)*,* genes in response to the 80 ng/ml rWNT3A, 50 ng/ml rRSPO1 and tetracycline overnight treatments in the parental, RNF43 and RNF43ΔPA (clone #1 and clone #2) TetON cell lines assessed by the qPCR. Data are presented as relative expression, 2^−ΔΔCt^ ± SD. **d.** Immunofluorescence of RNF43 and RNF43ΔPA intracellular localization. GFP with plasma membrane targeting signal served as plasma membrane marker (in green), HA tag for the detection of exogenous RNF43 and RNF43ΔPA mutant (in red). Nuclei stained with TO-PRO-3 Iodide are shown in blue. Scale bars represent 25 μm. Signals intensities were measured along selections and plotted. **e.** T-REx 293 cells overexpressing RNF43 were compared with the parental cell line for their ability to mediate TCF/LEF-dependent transcription in the Top flash dual luciferase assay. Response to 80 ng/ml rWNT3A, 25 ng/ml rRSPO1 and Tet treatments was measured after overnight incubation. Values are normalized to the unstimulated control cells. *N* = 4, unpaired two-tailed t-test **p < 0.01, ***p < 0.001. **f.** Western blot analysis of canonical Wnt pathway activation by application of the increasing concentrations of rWNT3A (40, 60 and 100 ng/ml) after 3 h of treatment. In cells overexpressing RNF43 phosphorylation of S1490-LRP6 and DVL2 (arrowheads) were weaker. Signal corresponding to the β-actin signal was used as loading control and HA tag specific antibody for RNF43 detection, *N* = 3

In order to address the role of PA domain of RNF43 we generated T-Rex 293 stable cell lines overexpressing human wild type (wt) RNF43 and RNF43 variant lacking its PA domain (Δ87–187), RNF43ΔPA, in a tetracycline (Tet)-controlled manner (TetON) (Fig. 1a). TetON system provided uniform RNF43 expression upon Tet addition, as shown by the western blot and quantitative real-time Polymerase Chain Reaction (qPCR) (Fig. 1a, b, c). Transgenic lines did not show altered expression of *ZNRF3*, encoding E3 ligase functionally redundant with RNF43, or *CTNNB1* encoding β-catenin (Fig. 1c’, c”). This suggests that we have not selected cell lines functionally compensating for the changed levels of RNF43.

Deletion of the PA domain could in principle harm protein processing, since RNF13 and RNF167 proteins lacking their PA fold were shown to have impaired cellular localization [30]. To address this issue, we performed the immunofluorescent imaging of cells expressing RNF43 variants and membrane-bound form of GFP, which served as the plasma membrane marker. Images and signals histograms confirmed that both wt RNF43 as well as RNF43ΔPA showed comparable subcellular localization and were present at the plasma membrane (Fig. 1d).

After initial and positive validation of our tools, we decided to move on to the functional assays. T-REx 293 cells represent a suitable cell line for the analysis of Wnt/β-catenin signal transduction and the role of RNF43 in this process. Prototypical ligand activating Wnt/β-catenin signaling cascade - rWNT3A triggered only a minor response when assessed by Top flash dual luciferase assay (Fig. 1e, left). However, once RNF43/ZNRF3 was inactivated by rRSPO1, rWNT3A activated β-catenin mediated transcription approx. 30x more efficiently (Fig. 1e, right). Importantly, Tet-induced exogenous RNF43 blocked cellular response to both rWNT3A and rWNT3A/rRSPO1 combination (Fig. 1e). Similar mechanism could be demonstrated by the western blot analysis of the key Wnt pathway proteins – namely phosphorylation of LRP6 at serine (S) 1490 and phosphorylation of DVL2 (detected as electrophoretic mobility) (Fig. 1f). Phosphorylation of LRP6 and DVL proteins mark canonical Wnt pathway activation [31, 32]. In agreement with the Top flash dual luciferase assay results, rRSPO1 synergized with rWNT3A and RNF43 overexpression suppressed activation of the LRP6 and DVL2 after 3 h long rWNT3A stimulation (Fig. 1f). It is also worth noting that RNF43 TetON cells without Tet induction also exhibited reduced pS1490-LRP6 signal; this is probably due to the TetON promoter leakage that is however below our detection limits (see anti-HA in Fig. 1f).

### RNF43ΔPA inhibits canonical Wnt signaling pathway

**Fig. 2:**
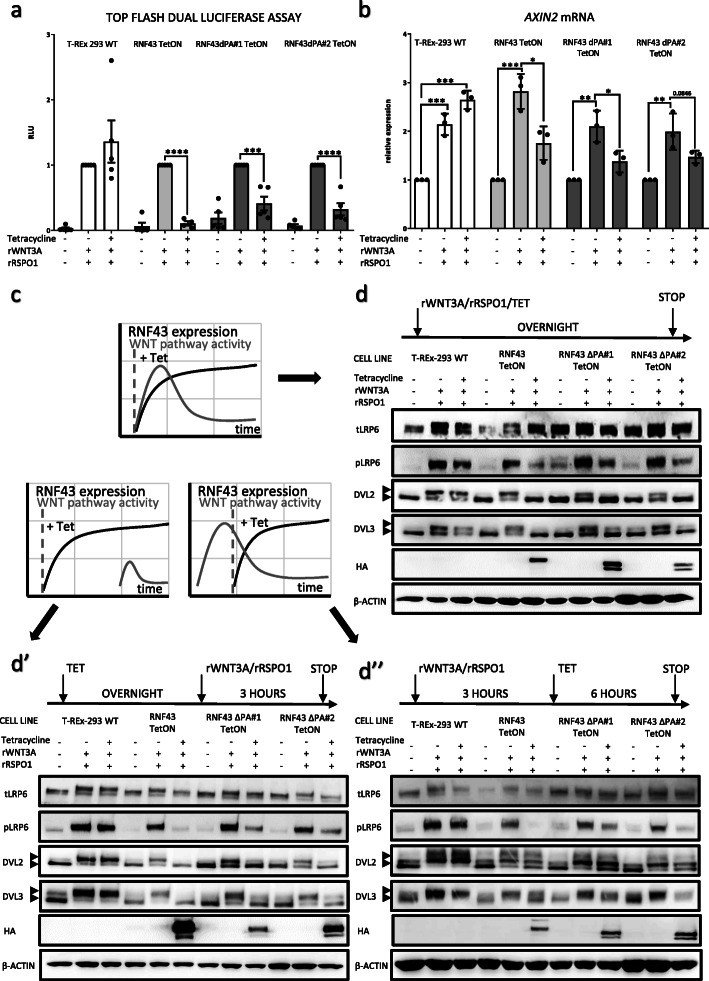
RNF43ΔPA inhibits canonical Wnt signaling pathway. **a**. Top Flash dual luciferase assay performed in the T-REx 293, RNF43 TetON and two RNF43ΔPA TetON cell lines in the response to the 100 ng/ml rWNT3A and 50 ng/ml rRSPO1 treatments in the presence of 0.5 μM LGK-974. Both RNF43 variants efficiently inhibited cellular responses. Results were normalized to Tet free conditions. N = 4, unpaired two-tailed t-test ***p < 0.001, ****p < 0.0001. **b.** Expression of the canonical Wnt signaling target gene *AXIN2* in response to the rWNT3A (100 ng/ml) and rRSPO1 (50 ng/ml) treatments. Cells overexpressing RNF43ΔPA mutant, similarly to the wild type RNF43, showed weaker *AXIN2* expression. Results were normalized to the assay values of the unstimulated samples. *N* = 3, unpaired two-tailed t-test. *p < 0.05, **p < 0.01, *** < 0.001. Data are presented as 2^−ΔΔCt^ ± SD. **c.** Schematic representation of performed experiments elucidating an impact of RNF43ΔPA on the different canonical Wnt signaling modes of activity – Wnt pathway activation and RNF43 constructs expression simultaneously, before and after pathway stimulation. **d.** Western blot analysis of RNF43ΔPA impact on the canonical Wnt pathway components. Phosphorylation of S1490 of LRP6 together with the DVL2 and DVL3 activation manifested by electrophoretic, phosphorylation specific shifts (arrowheads) upon RNF43 or RNF43ΔPA tetracycline forced expression along the 80 ng/ml rWNT3A and 25 ng/ml rRSPO1 treatments (d), before them (d’) or after (d”) stimulations. Both variants of RNF43 showed inhibitory effect on the β-catenin dependent Wnt signaling in the all tested conditions. LGK-974 was used to inhibit autocrine production of the Wnt ligands, HA tag signal corresponds to the RNF43 proteins and β-actin was employed as loading control

As a next step we took advantage of the model system described in Fig. 1 and started to analyze functionally the role of RNF43 PA domain. Firstly, we compared RNF43 ΔPA mutant with wt RNF43 in their capacity to inhibit rWNT3A and rRSPO1-induced β-catenin dependent transcription. Surprisingly, deletion of PA domain had no impact on the ability to down-regulate Top flash dual luciferase assay signal in both tested RNF43ΔPA TetON cell lines (Fig. 2a). To further back up the reporter assay results, we analyzed the expression of the Wnt/β-catenin pathway target gene *AXIN2.* Both RNF43 and RNF43ΔPA overexpression reduced *AXIN2* expression level comparably (Fig. 2b).

Finally, we have analyzed the effects of RNF43 variants on Wnt pathway activation using the set of Western blot readouts – namely phosphorylation of LRP6, DVL2 and DVL3. This experiment was performed in three setups: (i) RNF43 expression and Wnt pathway was induced at the same time, (ii) RNF43 expression preceded activation of Wnt pathway and (iii) RNF43 was induced when Wnt pathway was already activated (for schematics see Fig. 2c). These experimental variants addressed different properties of RNF43 – namely the potential to prevent the switch ON of the Wnt pathway (in i and ii) and potential to induce switch OFF (in iii) (Fig. 2c). Nevertheless, Tet-driven expression of RNF43 wt and RNF43ΔPA in all cases averted cellular responses to the rWNT3A/rRSPO1 and efficiently reduced phosphorylation of LRP6 as well as of DVL2 and DVL3 (Fig. 2d, d’, d”). These results suggest that RNF43ΔPA, similarly to the wild type RNF43, desensitized cells to the Wnt ligand stimulation and inhibited already activated receptor complexes.

### RNF43 ΔPA efficiently rescues phenotype of RNF43/ZNRF3 dKO cells

**Fig. 3:**
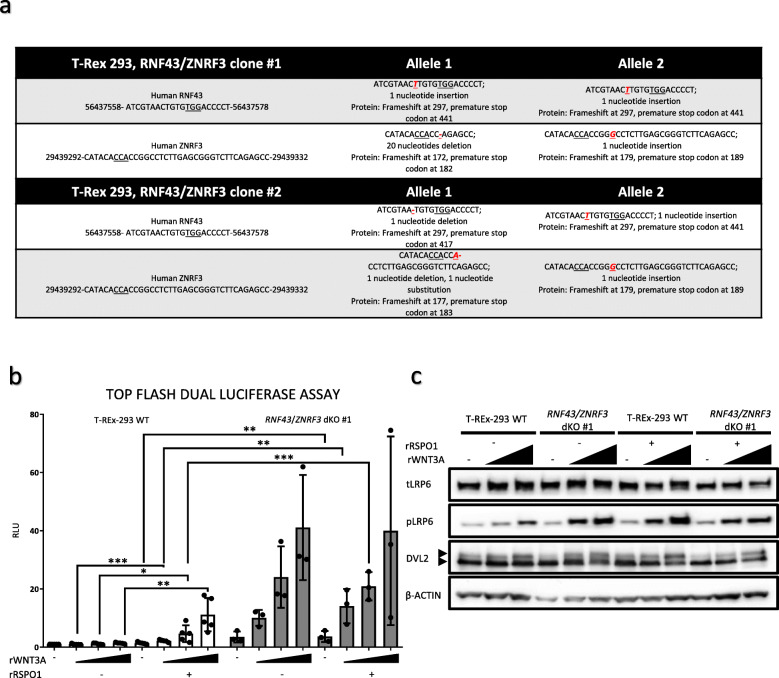
Preparation of cells lacking functional RNF43/ZNRF3 proteins. **a.** In order to generate double knockout lines, CRISPR/Cas9 method was applied to introduce mutations in the *RNF43* and *ZNRF3* genes of T-REx 293 cells. Sequencing results of *loci* targeted by the CRISPR/Cas9 approach. PAM sequences are underlined. Modifications effects on the protein sequence were predicted. **b.***RNF43*/*ZNRF3* dKO cells had higher β-catenin dependent transcriptional activity in response to the overnight incubation with different rWNT3A doses (40, 60 and 80 ng/ml). Also, dKO cell line was insensitive to the 25 ng/ml rRSPO1 treatment. All values are normalized to the unstimulated control cells results. *N* = 5 (wild type cells), *N* = 3 (dKO), unpaired two-tailed t-test *p < 0.05, **p < 0.01, *** < 0.001. **c.** Western blot analysis of canonical Wnt pathway activation in response to the increasing doses of rWNT3A (40, 60 and 100 ng/ml) after 3 h long treatments. *RNF43*/*ZNRF3* dKO exhibited stronger response to the rWNT3A in comparison to the parental cells and rRSPO1 stimulation had no effect. Signal corresponding to the β-actin signal was used as loading control, *N* = 3

The current experiments shown in Figs. 1 and 2 used the overexpression system that produced high RNF43 levels (see Fig. 1c). In order to analyze RNF43 activity at the physiological level, we generated T-REx™-293 cells lacking functional RNF43/ZNRF3 proteins by employing the CRISPR/Cas9 method (Clustered Regularly Interspaced Short Palindromic Repeats/Cas9). We have characterized in detail two *RNF43/ZNRF3* double knockout (dKO) cell lines, which were confirmed by sequencing of targeted genomic loci and prediction of modification consequences on the protein level (Fig. 3a). In line with the literature, *RNF43/ZNRF3* dKO cell line showed stronger response to rWNT3A in the Top flash dual luciferase assay, but in the rRSPO1-insensitive manner (Fig. 3b). Similar behavior was also confirmed by the western blot of the activation markers (Fig. 3c). Despite the fact that LRP6 is the reported target of RNF43/ZNRF3 [10, 11], our cells do not show alteration in global LRP6 levels, a result similar to the work of Jiang et al. [13]. It is thus possible that the consequence of the LRP6 ubiquitination is primarily the internalization of LRP6.

**Fig. 4:**
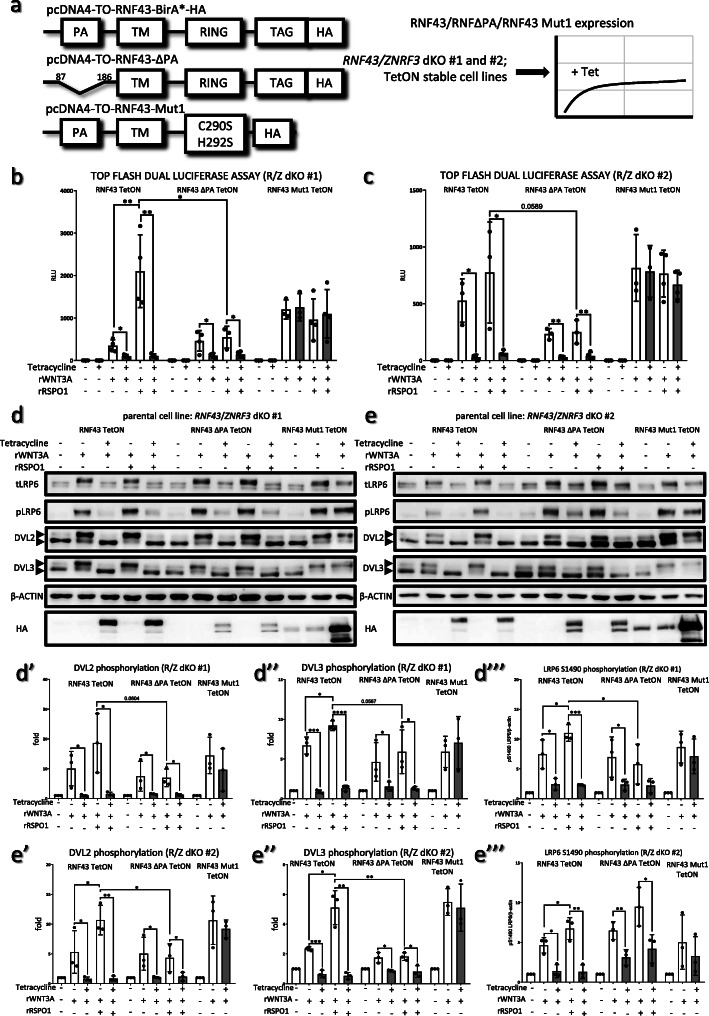
Both RNF43 and RNF43ΔPA rescue phenotypes of *RNF43/ZNRF3* dKOs cells. **a.** pcDNA4 plasmids encoding RNF43 wt, RNF43ΔPA and enzymatically inactive RNF43 Mut1 were transfected into CRISPR/Cas9 derived *RNF43*/*ZNRF3* dKO #1 and #2 T-Rex cell lines. Monoclonal stable TetON cell lines were derived thanks to antibiotic selection and colonies picking. **b., c.** Top flash dual luciferase assay in the *RNF43*/*ZNRF3* dKO #1 (**b**) and #2 (**c**) derivates expressing RNF43 wt, RNF43ΔPA or RNF43 Mut1 in the tetracycline sensitive way. Cells in all conditions were treated with the LGK-974. Tetracycline induced expression of RNF43 and its variants, recombinant WNT3A (80 ng/ml) activated canonical Wnt signaling and rRSPO1 (100 ng/ml) co-treatment was used to antagonize RNF43 action. Results were normalized to the untreated samples values and compared using unpaired t-test *p < 0.05, **p < 0.01 *N* = 3 or 4. **d., d’., d”., d”’, e., e’., e”. e”’.** Top flash samples were also analyzed by the Western blot for further investigation of the Wnt signaling activity in presence of different RNF43 variants in the *RNF43*/*ZNRF3* dKO #1 (**d**) and dKO #2 (**e**). Tetracycline forced expression of both RNF43 wt and RNF43 RNF43ΔPA, but not RNF43 Mut1, suppressed LRP6 1490S, DVL2 and DVL3 phosphorylation events. Recombinant RSPO1 treatment antagonized RNF43 wt, but not RNF43 lacking PA domain. RNF43 lacking enzymatic activity (Mut1) had no effect on the canonical Wnt signaling. DVL2 (d’, e’), DVL3 (d”, e”) and LRP6 (d”’, e”’) activation was quantified by the ImageJ software and presented as ratios of phosphorylation specific upper band to the lower one (arrowheads). Results were normalized to the untreated samples values and compared by t-test, *N* = 3 *p < 0.05, **p < 0.01, *** *p* < 0.001, **** *p* < 0.0001

*RNF43/ZNRF3* dKO cells, which are hypersensitive to the rWNT3A treatment, allowed us to perform complementation assays with RNF43 wt and RNF43ΔPA. In these rescue experiments RNF43 wt, RNF43ΔPA and enzymatically inactive RNF43 Mut1 carrying point mutations in the RING domain (negative control) were introduced to both *RNF43*/*ZNRF3* #1 and #2 dKOs under the control of tetracycline (TetON), granting the generation of clonal inducible stable cell lines (Fig. 4a).

Top flash dual luciferase assay performed in the TetON *RNF43*/*ZNRF3* #1 and #2 dKOs, confirmed that RNF43 lacking PA domain efficiently inhibited canonical Wnt signaling (Fig. 4b and c). Induction of RNF43 as well as RNF43ΔPA mutant was also capable to efficiently suppress WNT3A-induced S1490 phosphorylation of LRP6 and activation of DVL2 and DVL3 (Fig. 4d-e). Finally, expression of enzymatically dead RNF43 Mut1 variant incapable to perform ubiquitination reaction had no effect on the Wnt signaling (Fig. 4b-e”’), which suggest that the observed RNF43-mediated phenotypes are specific.

Interestingly, treatment with recombinant R-spondin1 (RSPO1) was unable to revert the inhibitory action of Tet-induced RNF43 and RNF43 ΔPA (Fig. 4b-e). However, we observed the induction of Wnt pathway by rWNT3A– determined as Top flash activity, DVL2, DVL3 and LRP6 phosphorylation - by combined rRSPO1 treatment in the RNF43wt, but not RNF43ΔPA and RNF43 Mut1, *RNF43*/*ZNRF3* dKO #1 and #2 cells in the absence of Tet (Fig. 4b-e). Upon closer inspection we found that leakage of TetON system leads to low expression of the transgene even in the absence of tetracycline (see HA signal in the Fig. 4d and e for RNF43 Mut1 results and Additional file 1 4d and 4e long expositions of HA signal). We propose that under these conditions RSPO1 treatment inhibits wt RNF43 but not RNF43 ΔPA.

We conclude that depletion of the PA domain produced variant insensitive to the negative regulation by rRSPO1 protein, what would be in agreement with published crystal structures showing direct interaction between PA domain of RNF43 and Furin-1 fold of R-spondins, ultimately leading to the internalization of RNF43 [15]. In summary, using multiple sets of assays we provide evidence that the extracellular PA domain is not essential for RNF43 inhibitory activity in the Wnt/β-catenin pathway in human cells but rather it serves as a module that via action of RSPO1 regulates surface levels of RNF43.

## Discussion

Our experiments conclude that in mammalian cells PA domain of RNF43 does not contribute to the ability of RNF43 to efficiently inhibit WNT3a-induced β-catenin-dependent signal transduction. Namely, RNF43ΔPA was comparably potent as wt RNF43 in (i) the inhibition of Top flash dual luciferase assay and *AXIN2* gene expression, (ii) block of WNT3a-induced phosphorylation of LRP6 and (iii) phosphorylation of DVL2 and DVL3. We demonstrated this capacity using inducible expression of RNF43ΔPA as well as by rescue experiments in *RNF43/ZNRF3* dKO cells. Notably, in the rescue experiments RNF43ΔPA showed significantly stronger inhibitory effect on the pS1490 LRP6 than wt RNF43. We propose that in this experimental setup RNF43ΔPA variant cannot be regulated by the endogenously secreted RSPOs that bind via PA domain [2] and as such can have enhanced activity. This was confirmed in the Top flash dual luciferase assay, by which we showed that RNF43ΔPA was not sensitive to the recombinant RSPO1 treatment.

Our key results suggest that the key function of the PA domain of RNF43 in human cells is to mediate negative regulation by R-spondin family proteins. This is in contrast to some of the previously published data that propose a role of PA domain in the inhibition of Wnt pathway. We see multiple reasons possibly explaining existing discrepancies. We discuss individual aspects namely: (i) studies in non-vertebrate models, (ii) biochemical studies on (RNF43) PA and (FZD) CRD domains, (iii) studies performed in vertebrates and (iv) presence of RNF43 PA domain mutations in Wnt-addicted tumors in detail below.

One of the key studies about PA domain function was based on the *Caenorhabditis elegans* model organism. Nematode ortholog of human RNF43 and ZNRF3 proteins, PLR-1 blocked Wnt signaling and caused the endosomal internalization of Frizzled receptors (MIG-1 in *C. elegans*) in a manner dependent on Frizzled’s Cysteine Rich Domain (CRD) and RING and PA folds of PLR-1 [21]. This demonstrated the functional interaction between extracellular parts of PLR-1 and MIG-1. Interestingly, *C. elegans* lacks R-spondin related proteins. It was shown before that RSPO/LGR regulatory axis evolved only in vertebrates, so it is not present in *C. elegans* [33]. Thus, we can speculate that the role of the PA fold changed together with the evolutionary rise of the Wnt signaling complexity.

The other issue that remains partially unsolved is the physical interaction between PA domain of RNF43 and CRD of FZDs, as its existence was detected in the co-immunoprecipitation assays after overexpression PA and CRD domains [21, 22]. This still remains controversial, since two other groups were not able to detect direct interaction between RNF43 and Frizzled by structural biology approaches [13, 18]. During immunoprecipitation, the interaction could be indirect and could include other proteins such as endogenously expressed R-spondins. Further report showed that RNF43 lacking the whole extracellular part was not able to promote FZD5 internalization [10]. However, it seems to us that this particular RNF43 mutant lacked signal peptide, and as such could not be targeted to the plasma membrane. In our study RNF43ΔPA contained the whole sequence between signal peptide and PA part, which might be involved in the unknow protein-protein interactions.

The solid work by Tsakayuma and others [22] suggested that even in vertebrate cells the PA domain of RNF43 is important for its function. However, still the authors observed a weak but clear inhibitory activity towards Wnt/β-catenin pathway in vivo in *Xenopus* embryonic development. It suggests that PA domain is not essential but in the embryonal context RNF43ΔPA acts as a weaker inhibitor. We can only speculate if other RNF43 functions participate on the inhibitory action of RNF43 in this context. For example RNF43 was described to act downstream of plasma membrane receptor complexes and even downstream from mutated β-catenin by sequestering the Transcription factor 4 (TCF4) to the nuclear membrane and thereby, leading to the inhibition of gene expression regulated by this transcriptional factor [12].

It was reported that missense mutations (R127P and A169T) occurring within the PA domain suppressed inhibitory properties of RNF43 towards the canonical Wnt signaling [12, 22]. These genetic changes, together with others (i.e. K108E, R117H, E170K), are described in cases of large intestine and pancreatic cancers (COSMIC database: cancer.sanger.ac.uk [34]), suggesting *RNF43* loss-of-function. RNF43/ZNRF3 bind its endogenously produced inhibitors – R-spondins, exactly via the PA fold (residues Gln84, His86, Lys108, and Glu110) [16]. Consequently, mutations disrupting these interactions are expected to lead to the insensitivity of RNF43 towards R-spondins, which is counterintuitive in cases of these Wnt-addicted tumors. Therefore, an alternative explanation is that these mutations affect folding and/or localization of RNF43 [12, 22] or affect binding to other, not known yet RNF43 interactors. The misfolding/misplacement hypothesis is supported by the importance of PA domains in other PA-TM-RING E3 ubiquitin ligases where PA domains serve as motives for the protein-protein interactions [35] and subcellular localization [30]. For example, PA domain of RNF13 and RNF167, from the same group of E3 ligases as RNF43/ZNRF3, was indispensable for their homodimerization and proper cellular localization [30]. In line with that R127P variant showed impaired reticular expression in contrast to the wild type protein and possible ERAD-mediated degradation [22]. It is thus probable that the analysis of the biological output of such mutants tells us only a little about the physiological function of the PA domain.

## Conclusion

In summary, we showed by the biological assays and precise modulation of protein levels that RNF43 lacking PA domain can still efficiently inhibit cellular events related to the canonical Wnt signaling pathway. In some assays RNF43ΔPA mutant was even a better inhibitor which points to the importance of PA domain as the primary regions required for the RNF43 regulation by R-spondin 1. Altogether, our study highlights a necessity for further work aiming to the better description of still quite poorly understood mechanisms of regulation and function of RNF43 and ZNRF3 enzymes in the homeostasis and pathology.

## Supplementary information

**Additional file 1.**

## Data Availability

The datasets used and/or analyzed during the current study are available from the corresponding author on reasonable request.

## Abbreviations

CRD: Cysteine-rich domain (CRD)
CRISPR/Cas9: Clustered Regularly Interspaced Short Palindromic Repeats/Cas9
CTNNB1: Beta-catenin
DMEM: Dulbecco’s modified Eagle’s medium
DVL: Dishevelled
FBS: Fetal bovine serum
FZD: Frizzled
HA: Hemagglutinin tag
LGR4–6: Leucine-rich repeat-containing G-protein coupled receptor 4–6
LRP5/6: Low-density lipoprotein receptor-related protein 5/6
PA: Protease-associated
PAM: Protospacer adjacent motif
PCR: Polymerase chain reaction
PEI: Polyethylenimine
qRT-PCR: Quantitative real-time polymerase chain reaction
RING: Really Interesting New Gene
RNF43: RING finger protein 43
RSPO1–4: R-spondin 1–4
SD: Standard deviation
TCF4: T-cell factor 4
TET: Tetracycline
TetON: Tetracycline-controlled transcriptional activation
ZNRF3: Zinc/Ring finger protein 3
