# Supplementary figures and images for: Protease associated domain of RNF43 is not necessary for the suppression of Wnt/β-catenin signaling in human cells
Source: Cell Commun Signal. 2020 Jun 11;18:91. doi: 10.1186/s12964-020-00559-0 (PMC7291719; doi:10.1186/s12964-020-00559-0)

**Fig. 1 f**

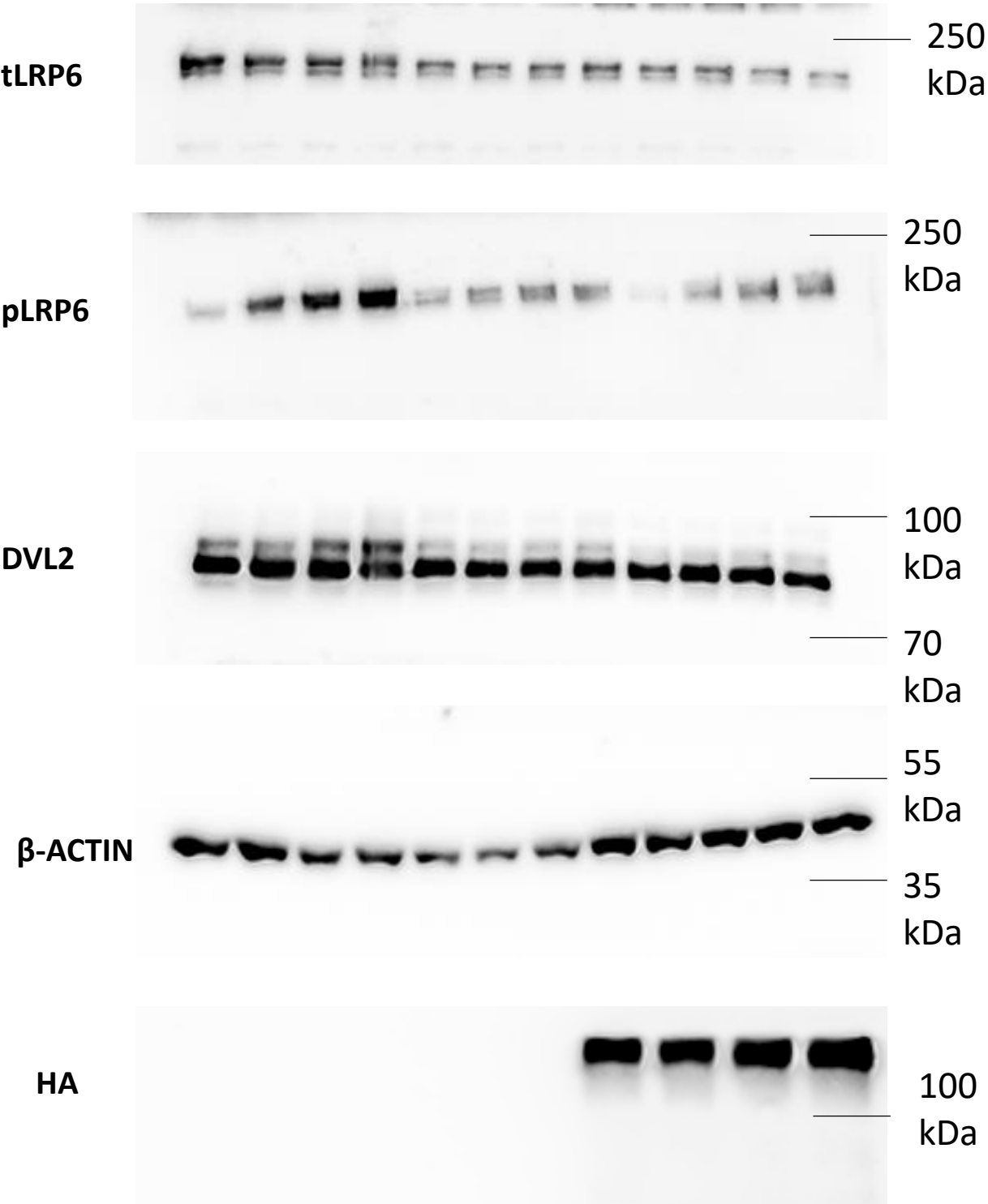

**Fig. 2. d**

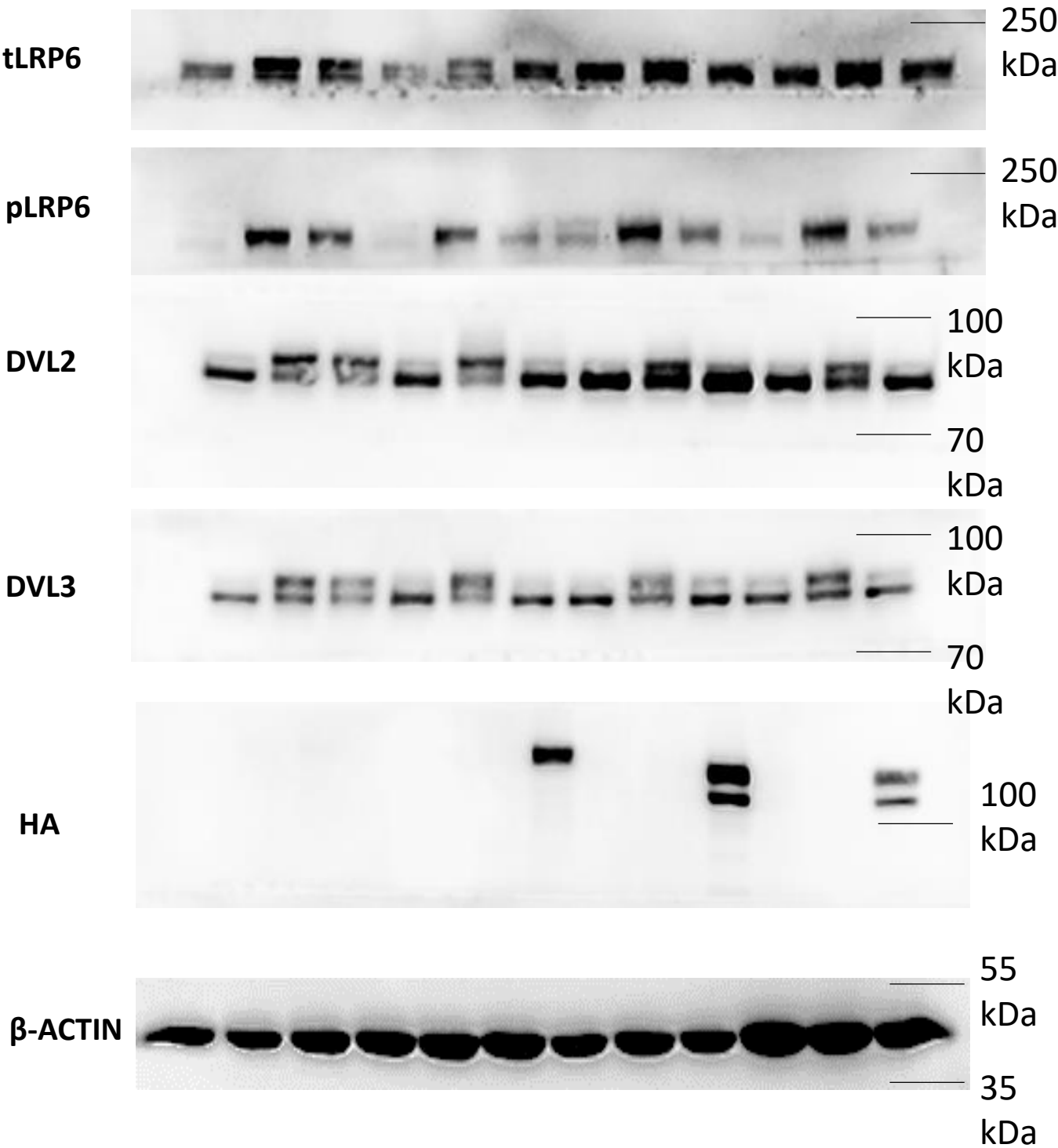

**Fig. 2 d'**

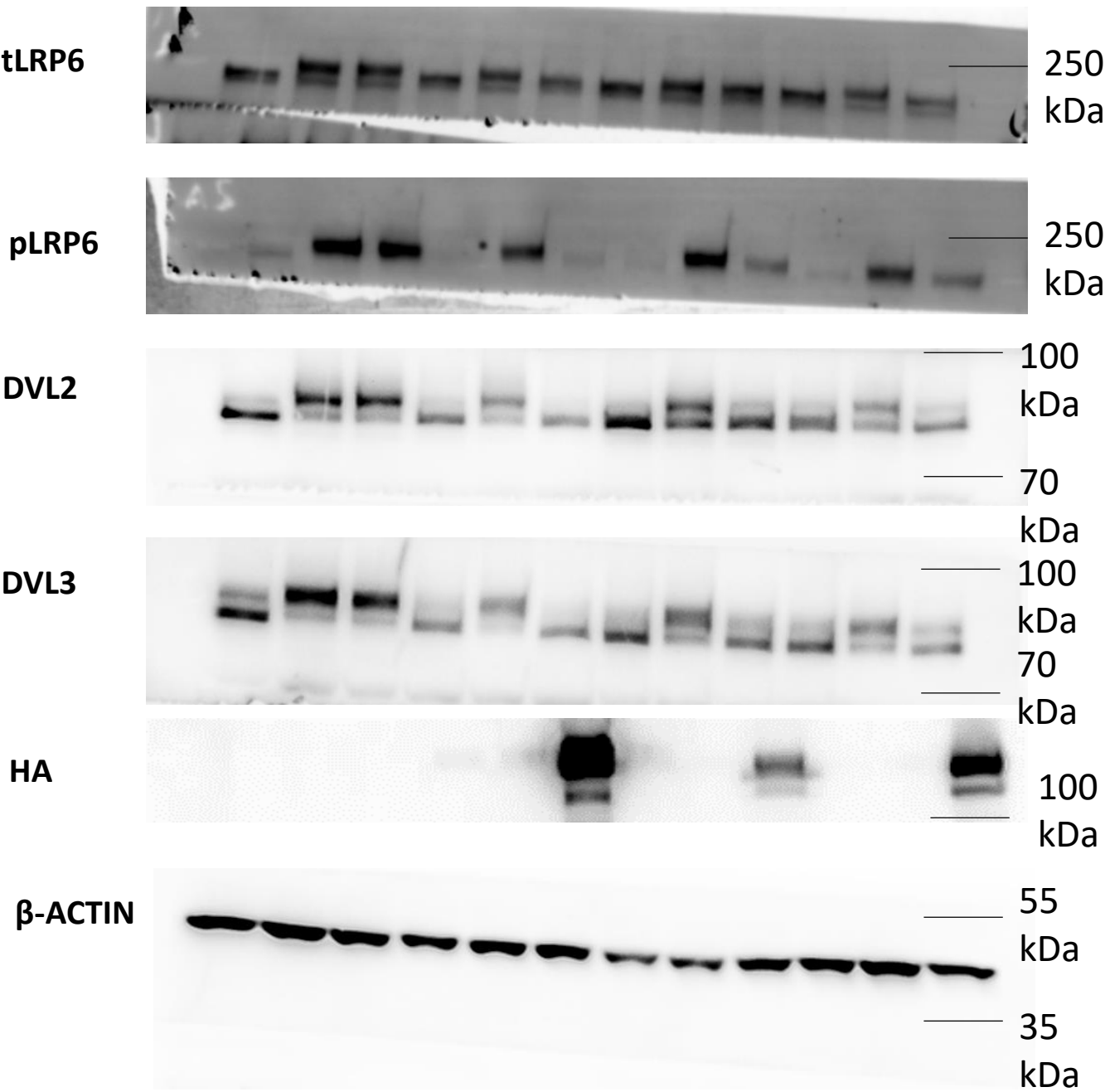

**Fig. 2 d''**

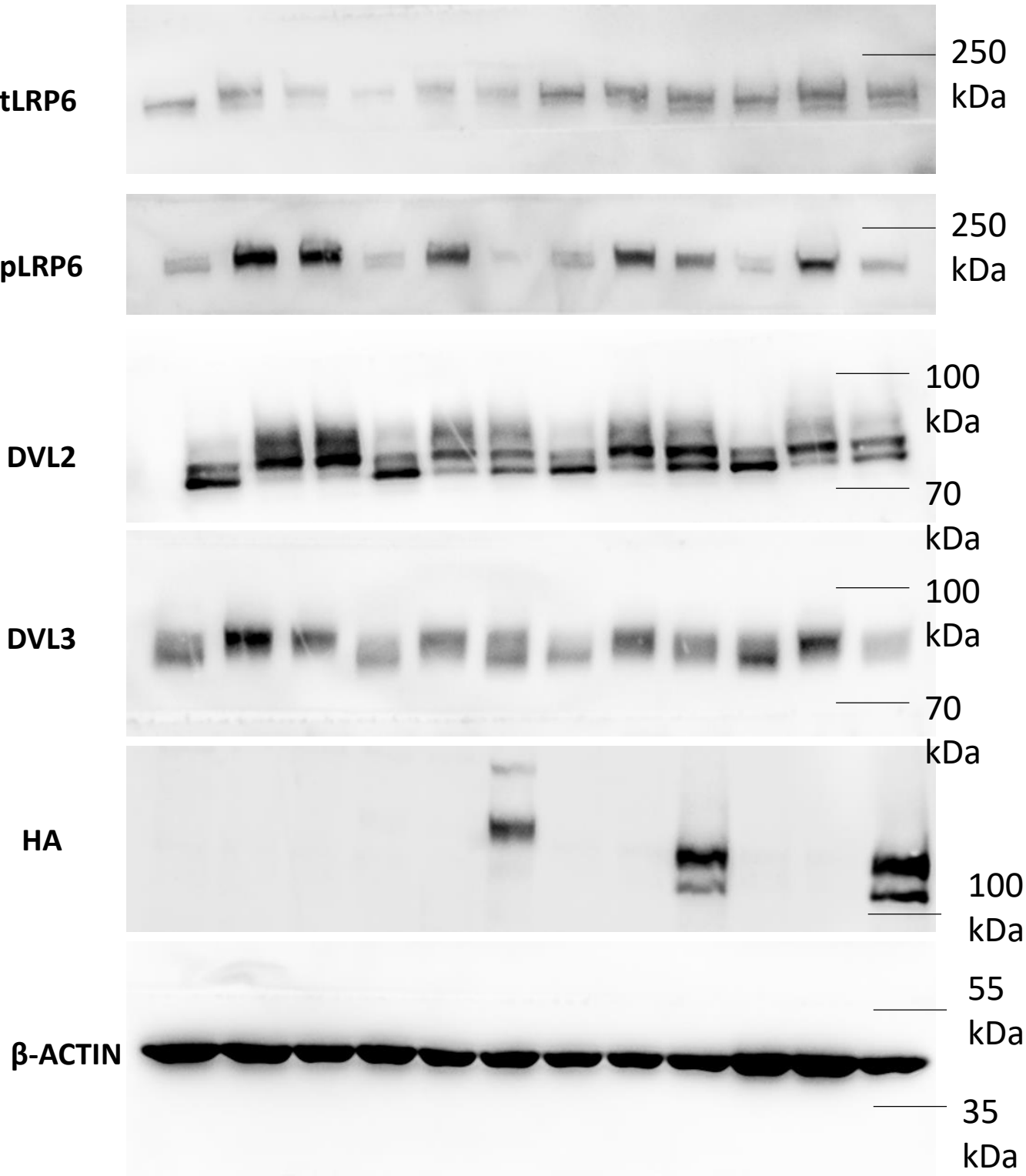

**Fig. 3 c**

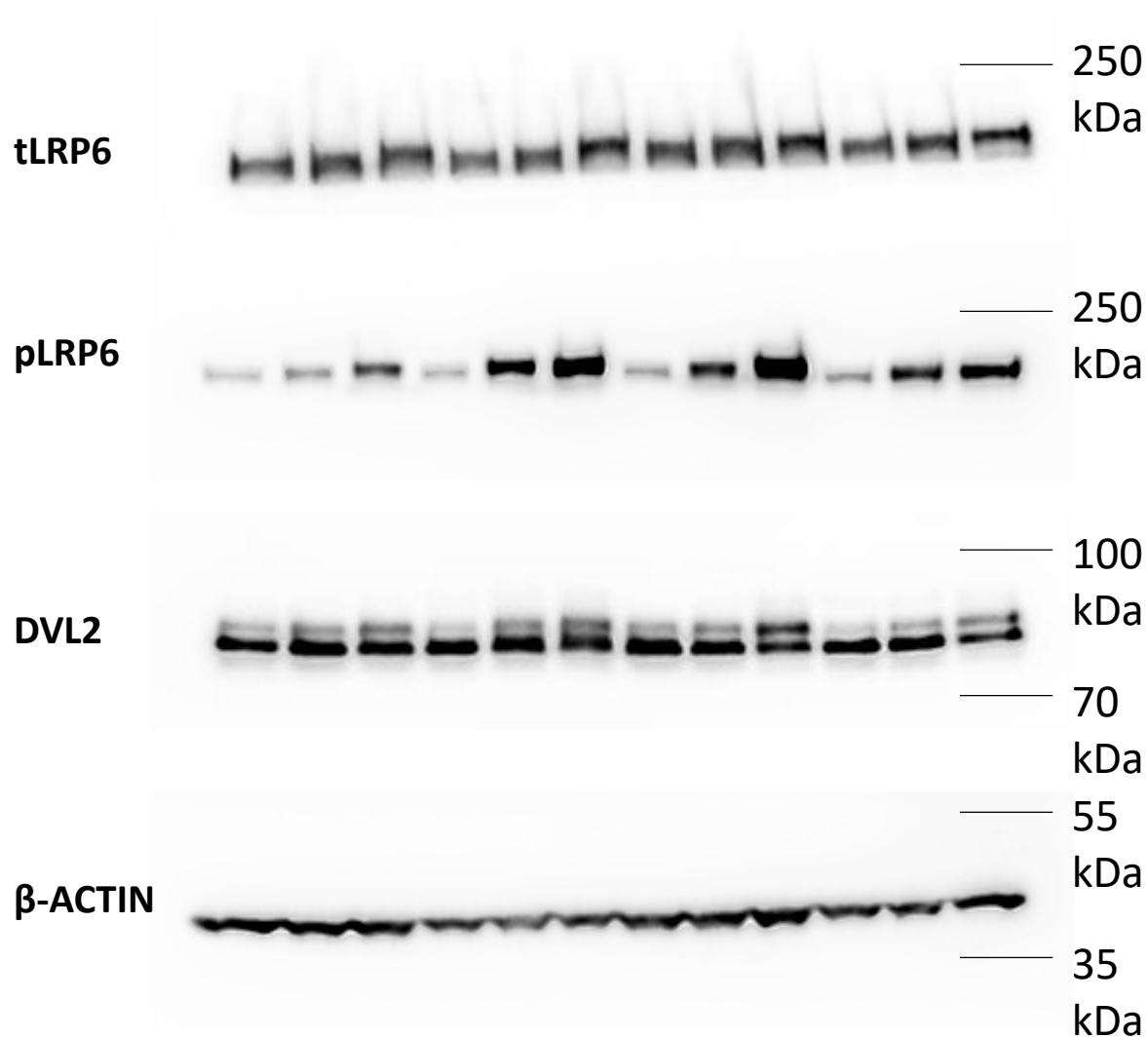

**Fig. 4 d**

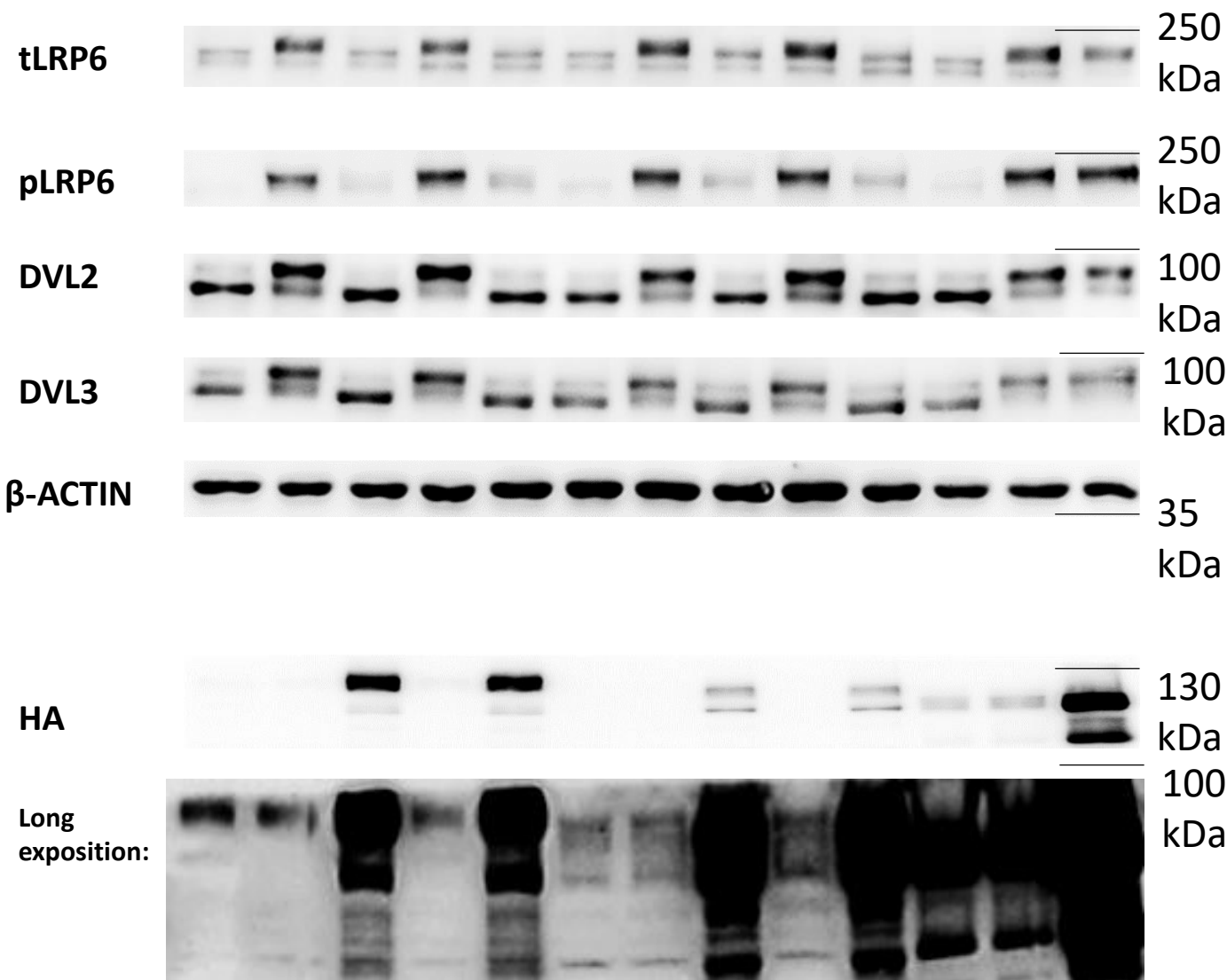

**Fig. 4 e**

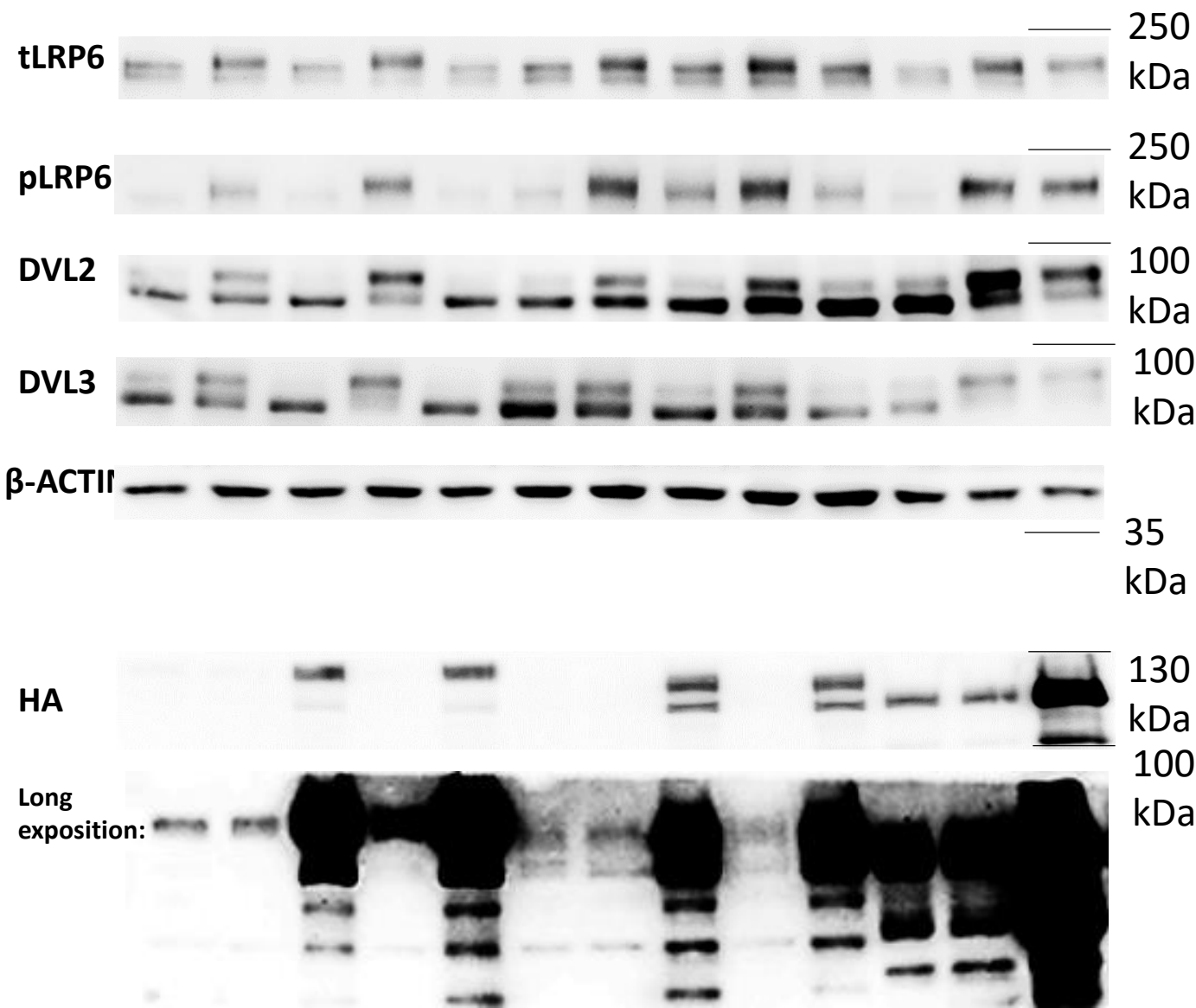

Supplement: Supplementary file 2 — Additional file 1. [file 12964_2020_559_MOESM2_ESM.pdf]
